# Stem cell therapy: a new hope for stroke and traumatic brain injury recovery and the challenge for rural minorities in South Carolina

**DOI:** 10.3389/fneur.2024.1419867

**Published:** 2024-08-09

**Authors:** Ghada A. Mohamed, Daniel H. Lench, Parneet Grewal, Mark Rosenberg, Jenifer Voeks

**Affiliations:** Department of Neurology, Medical University of South Carolina, Charleston, SC, United States

**Keywords:** stem cells, stroke, traumatic brain injury, disparities, South Carolina

## Abstract

Stroke and traumatic brain injury (TBI) are a significant cause of death and disability nationwide. Both are considered public health concerns in rural communities in the state of South Carolina (SC), particularly affecting the African American population resulting in considerable morbidity, mortality, and economic burden. Stem cell therapy (SCT) has emerged as a potential intervention for both diseases with increasing research trials showing promising results. In this perspective article, the authors aim to discuss the current research in the field of SCT, the results of early phase trials, and the utilization of outcome measures and biomarkers of recovery. We searched PubMed from inception to December 2023 for articles on stem cell therapy in stroke and traumatic brain injury and its impact on rural communities, particularly in SC. Early phase trials of SCT in Stroke and Traumatic Brain injury yield promising safety profile and efficacy results, but the findings have not yet been consistently replicated. Early trials using mesenchymal stem cells for stroke survivors showed safety, feasibility, and improved functional outcomes using broad and domain-specific outcome measures. Neuroimaging markers of recovery such as Functional Magnetic Resonance Imaging (fMRI) and electroencephalography (EEG) combined with neuromodulation, although not widely used in SCT research, could represent a breakthrough when evaluating brain injury and its functional consequences. This article highlights the role of SCT as a promising intervention while addressing the underlying social determinants of health that affect therapeutic outcomes in relation to rural communities such as SC. It also addresses the challenges ethical concerns of stem cell sourcing, the high cost of autologous cell therapies, and the technical difficulties in ensuring transplanted cell survival and strategies to overcome barriers to clinical trial enrollment such as the ethical concerns of stem cell sourcing, the high cost of autologous cell therapies, and the technical difficulties in ensuring transplanted cell survival and equitable healthcare.

## Introduction

Stroke is the 6^th^ leading cause of mortality and a leading cause of morbidity in South Carolina and resulted in healthcare expenses of $1.3 billion in 2020 alone (1). The incidence of stroke varies by age, gender, race, and ethnicity. African American (AA) men are particularly vulnerable and have a 49 percent greater likelihood of dying from stroke than Caucasian Americans (CA) (2). A higher prevalence of stroke risk factors among AA and males compared to CA and females contributes to these disparities (2). A National Study of inpatient rehabilitation after the first stroke showed that AAs were younger and more disabled on admission, more likely to be discharged home and less likely to report independence on ADLs (3). Data from the Brain Attack Surveillance in Corpus Christi (BASIC) project also show that post-stroke Hispanic Americans scored worse on neurological, functional, and cognitive outcomes than CA (4).

Traumatic Brain Injury (TBI) is a significant cause of death and disability among the young population and is estimated to occur every 15 s in the United States (5). The economic impact of TBI is staggering, accruing an annual cost of over $77 billion in the United States (6). Between 2016 and 2018 about 4,310 TBI-related deaths were reported in South Carolina; this is 57.8% higher than the national average (7). These deaths were a combination of accidental, homicidal and suicidal causes. While the exact description of poor in South Carolina is unknown, health and economic barriers in this state may be more common than elsewhere. The degree of rurality played a role in higher incidences of TBI and increased barriers to emergency medical care (8). Racial and ethnic differences are apparent in acute and post-concussive management (9). During the early acute phase, there is a discrepancy in those taken to the hospital for evaluation (9). Afterward, there is a high risk of inadequate follow-up and management in the post-concussion period (10).

In addition, race and gender disparities in stroke and TBI care also play a significant role in patient outcomes. A report by the National Institute of Neurological Disorders and Stroke (NINDS) reveals that only 42% of the total population in clinical trials from 1985 to 2008 were women in acute stroke clinical trials (11, 12). Numerous studies report reduced access to emergency stroke care, delayed hospital arrivals, and limited rehabilitation resources for AA compared to CA (13). These disparities are echoed in clinical trials, with non-White minorities significantly underrepresented, which affects the validation and generalizability of clinical trial outcomes (11).

Treatment options for acute ischemic stroke are approved by the Food and Drug Administration (FDA) including intravenous thrombolytics (IVT) and mechanical endovascular thrombectomy (MT) (14). However the time-sensitive nature and strict selection criteria often exclude acute stroke patients from receiving these treatments. After stroke completion, dedicated rehabilitation for survivors is the only option with a proven long-term patient benefit (15). Of those who develop motor weakness after stroke, only 50% achieve functional independence at 6 months. Maximum rehabilitation benefit occurs within the first months after stroke (16). Similarly treatment options for TBIs outside neurosurgical intensive care units are limited. Lifestyle modifications, medication management, cognitive rehabilitation, and surgeries have been explored with mixed results (17).

**Figure 1:**
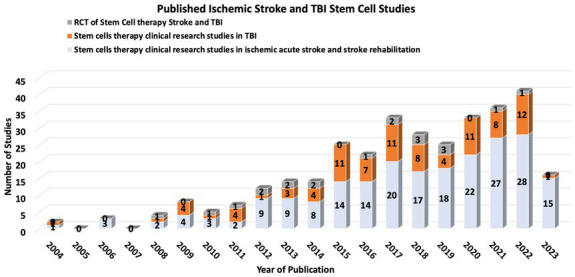
Number of published Stroke and TBI from 2014-2023.

Stem cell therapy (SCT) has emerged as a potentially transformative intervention for ischemic stroke and TBI, with the ambitious aim of replacing or aiding the recovery of neurons and vascular cells affected by ischemic events. While there are no current FDA-approved SCT trials for stroke or TBI, increasing research over the past decade shows some promising trends (18–20) (Figure 1).

## Stem cells in stroke and TBI clinical trials

Stem cell therapy is a potentially transformative intervention for ischemic stroke and TBI. Several clinical trials have addressed the utility of different stem cell types in ischemic stroke and TBI, including mesenchymal stem cells (MSCs), neural stem cells (NSCs), and induced pluripotent stem cells (iPSCs) (20, 21). These trials vary widely in the design of stem cell sources, dosages, delivery routes, and timing of post-stroke therapy.

Results of early-phase SCT clinical trials present a promising safety profile, with no significant adverse effects directly attributable to the therapy (22). Some trials have shown improvements in neurological function and reductions in lesion volume, but these findings have yet to be consistently replicated across a spectrum of studies. The Stem Cell Therapies as an Emerging Paradigm in Stroke (STEPs) committee has been formed to guide and bridge the gap between basic and clinical studies (23).

One noteworthy example is the multipotent adult progenitor cells in acute ischemic stroke (MASTERS) clinical trial, a phase 2 study exploring multipotent adult progenitor cells (MAPCs) in acute ischemic stroke (24). This trial enrolled 129 patients, allocating them to either a low or high dose of the cells or a placebo. While the treatment was deemed safe, no significant differences were observed in global recovery.

Stem cell therapy also may represent a breakthrough for stroke survivors, especially when combined with rehabilitation therapy (25). The two most extensive Randomized controlled trials (RCTs) for stem cell therapy in stroke rehabilitation and recovery in the US evaluated the impact of MSC in patients with stroke more than 6 months prior with safety endpoints and functional recovery endpoints. Both trials showed safety, feasibility and improved functional outcomes (26).

## Stem cells and outcome measures

**Figure 2:**
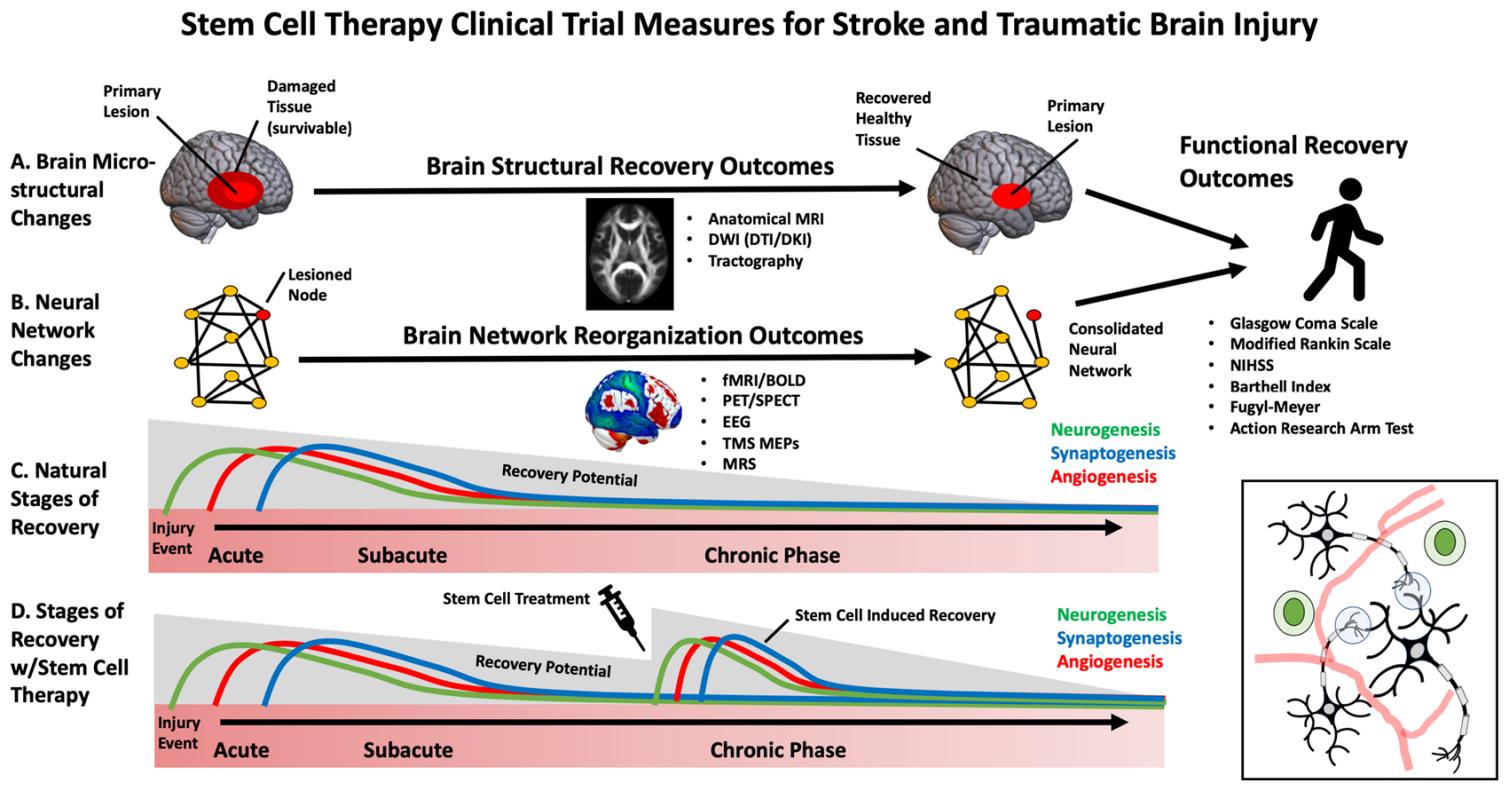
Diagram of stem cell effects on brain injury recovery and outcome measures.

While early clinical trials for SCT in stroke have primarily focused on feasibility and safety, some studies have begun to evaluate efficacy (27). Selecting the appropriate patients and outcome measures to maximize stem-cell clinical trials, sensitivity, specificity and power is necessary. This is especially important in stroke and TBI, in which heterogeneous brain circuitry is affected, and plasticity is highly dynamic throughout various stages of the recovery process (e.g., acute, subacute, chronic) (28). The most frequently used outcome measures in stem cell clinical trials for stroke and TBI have included broad, domain-general actions of disability, such as the Glasgow Coma Scale (GCS), modified Rankin Scale (mRS), National Institutes of Health Stroke Scale (NIHSS) (29), European Stroke Scale (ESS) and Barthell Index (BI). These measures address broad aspects of functional impairment but lack specificity. Domain-specific outcome measures include the Fugl Meyer Assessment (FMA), Action Research Arm Test (ARAT), and performance on specific functional tasks. These measures may provide more targeted, sensitive measures of behavioral change (30) (Figure 2).

## Biomarkers and mechanistic measures of brain recovery

While several stem-cell trials have focused on functional clinical outcome measures, there is an additional need to establish reliable biomarkers and mechanistic outcomes that capture brain-based changes during recovery (27). This enables effective translation between pre-clinical animal models and humans, allowing for more individualized and practical approaches to SCT (31). This is particularly important for disparities in stem cell clinical trials as it overcomes issues associated with language and cultural barriers that influence the reliability of subjective measures (13).

Currently there are no standardized or validated biomarkers for stroke or TBI stem cell treatments, making it difficult to determine which are optimal for clinical trials (32). Blood-based biomarkers have been investigated to measure growth factors and inflammation (33) which appear to be influenced by stem cell treatments in preclinical and clinical trials. Neurotrophic factors that support the survival and growth of brain tissue were explored in previous studies and included nerve growth factor (NGF), glial-derived neurotrophic factor (GDNF), and brain-derived neurotrophic factor (BDNF). Meanwhile, vascular endothelial growth factor (VEGF) and fibroblast growth factors (FGF) have been investigated as they may reflect vascular and tissue remodeling following injury (34). Serum-based inflammatory biomarkers can reflect the anti-inflammatory effects of stem cells and include inflammatory cytokines interleukins (IL; e.g., IL-2, IL-4, IL-6, IL1-beta, IL1-alpha, IL-10), tumor necrosis factor (TNF) alpha, and interferon-gamma among several others (34). Which of these growth factors and inflammatory biomarkers are the most sensitive and clinically meaningful within the context of stroke and TBI rehabilitation has yet to be determined still a matter of ongoing research (35). In addition to blood-based biomarkers, advances in brain imaging and non-invasive brain stimulation may prove to be useful tools in developing novel biomarkers for SCT clinical trials. These measures can be focused on changes to the primary site of injury or remote modifications, including reorganizing brain circuits affected by the injury. Recent advances in clinical neuroscience make it possible to non-invasively assess biological features of the brain, including structural integrity and neurophysiology (36). These novel tools, including neuroimaging and non-invasive brain stimulation to probe neural circuits, may be helpful in (1) developing more individualized approaches to stem cell treatment, (2) as an approach to stratify those who are most likely to benefit from a given therapy and (3) to understand how and where the cells are integrating into specific circuits or networks (37).

### Brain imaging measures

Neuroimaging of the brain has undergone significant advancement over the past decades. These approaches measure the neural architecture and activity thought to underly functional recovery after stroke and TBI and may provide a more accurate measure of brain recovery than clinical assessment tools (38). Due to the non-invasive nature of these approaches and widespread accessibility across major medical centers, neuroimaging is one of the most widely used objective outcome measures for neurological clinical trials.

Structural neuroimaging can evaluate changes in the anatomical features of brain tissue, including volumetric measurements, morphology, and tissue microstructure. This has been primarily performed using MRI. Routine clinical scans including high-resolution T1 scans, diffusion-weighted imaging (DWI), susceptibility-weighted imaging (SWI) and T2 scans may be utilized to estimate gray and white matter volume, lesion volume, penumbra volume, and cortical gyrification indices and have been informative biomarkers in pre-clinical stem cell studies (39). These calculations can help monitor changes in lesion size and impact overall brain morphometry throughout the recovery period in future human trials (40). Meanwhile, advanced DWI sequences can track complex fiber pathways and detailed information about brain tissue microstructure (41). Early limitations associated with tractography derived from diffusion tensor imaging (DTI), such as complex fiber-crossing, have undergone rapid advancement with more sophisticated approaches, including diffusion kurtosis imaging (DKI) and constrained spherical deconvolution (CSD) (42). Many studies have examined how the integrity of the corticospinal tract measured using fractional anisotropy relates to motor impairment in the context of stroke (38). Although the precise biological correlates of these diffusion measures and their interpretation are still being investigated, these approaches hold promise as a sensitive measure of changes in the health of brain tissue in clinical trials with stem cell therapies.

There is a growing appreciation that brain injury and its functional consequences cannot simply be explained by damage to a single structure but rather by the connectivity of that structure to an integrated network (43). Neuroimaging is an effective tool to assess neural activity within these distributed brain networks. In the context of stroke and TBI, reorganization of neural networks may underly recover after rehabilitation (37). Thus, it will be essential to understand the impact of stem cell interventions on these large-scale networks. Determining neural activity-specific timescales and spatial resolutions for quantitative change provides a reliable measure of structural changes in the brain. Specific neuroimaging approaches can be tailored to brain assessments in the setting of stem cell infusion. Functional Magnetic Resonance Imaging (fMRI) and electroencephalography (EEG) are the most widely used approaches to study cortical networks. fMRI relies on an indirect measure of neural activation by assessing how blood oxygenation levels change over time. The resulting blood oxygen level-dependent signal (BOLD) is acquired by subtracting “resting state” activity from neural activity during or during task engagement. fMRI has been used to demonstrate neural plasticity within neural networks following brain injury (44). While less spatially precise, EEG can directly measure neuroelectric activity at a high temporal resolution which is easily scalable across medical centers. EEG may predict functional outcomes and may be correlated with mRS, the FM and the NIHSS (32). Transcranial Magnetic Stimulation (TMS) may be combined with other neuroimaging approaches to probe non-motor networks or with neurophysiological recordings to assess motor pathways. TMS motor evoked potentials (MEPs) have been used to assess corticospinal integrity following stroke and are a good prognostic indicator of the extent of functional recovery (45).

While the previously mentioned approaches may be effective at identifying network remodeling and neuroplasticity, these approaches cannot assess angiogenesis and neurogenesis associated with stem cell therapies. Imaging modalities such as Positron Emission Tomography (PET) can evaluate changes in vasculature and neuronal survivability by measuring regional cerebral blood flow (rCBF) and metabolic rate using radiotracers. Magnetic resonance spectroscopy (MRS) can also monitor changes in metabolite composition and concentration within brain tissue (46, 47). This approach may provide a surrogate marker for cellular repair mechanisms and metabolic changes in the recovery process.

## Stem cells therapy challenges

The current challenges of stem cell therapy for stroke and TBI are multifactorial and significant. First, the best source of MSCs for stroke treatment has yet to be established (48, 49). Most preclinical studies used MSCs from healthy, young donors and about half of the clinical studies used autologous MSC (50). Harvesting stem cells from donors, especially neural stem cells (NSCs) or embryonic stem cells (ESCs), raises ethical concerns as well as concerns regarding the viability and effectiveness of stem cells from different donor types. Harvesting stem cells from donors, especially neural stem cells (NSCs) or embryonic stem cells (ESCs). Ethical issues arise primarily from the use of ESCs, which involves the destruction of embryos, and the use of NSCs, which often require fetal tissue. These ethical concerns can hinder research progress and limit the availability of stem cells for clinical use (51, 52). Although the use of autologous MSC addresses this issue, MSC are costly and require several months for optimal production; this delays administration beyond desired treatment windows (49, 53). The optimal timing for MSC administration is controversial; while very early transplantation within 48 h is recommended, some studies suggest benefits even 1 month post-stroke (54) (Supplementary Table 1). The administration route presents another hurdle: systemic approaches like intravenous (IV) and intra-arterial (IA), compared to direct intrathecal (IC) approaches carry potential risks and benefits. For instance, IV administration may lead to pulmonary trapping of cells, whereas IC administration poses risks of infection and bleeding (55). Technical challenges include Tracking transplanted cells to ensure survival and overcoming potential immune rejection. Imaging modalities including magnetic resonance imaging (MRI), positron emission tomography (PET), single-photon emission computed tomography (SPECT/CT), or bioluminescence imaging (BLI) using green fluorescent protein-Luciferase (GFP-Luc) may be utilized for stem cells *in vivo* tracking (53). Safety concerns persist, especially the risk of undesirable tissue differentiation and oncogenesis, exacerbated when genetic manipulation or reprogramming is employed to augment MSCs, potentially causing unregulated cell proliferation. This is underscored by instances where stem cell transplants have induced tumorigenesis (56, 57). Additionally, the survival and integration of transplanted cells into the host tissue remain significant hurdles. The hostile post-stroke environment, characterized by inflammation and scarring, can impede the survival and integration of transplanted cells (58).

Outcome evaluation measures should be clear and unified for patients receiving therapy whether functional, quality of life or cognitive preclinical and clinical study endpoints and outcome measurement methods were heterogeneous. Patient selection and treatment costs are other significant issues. Many stroke patients have comorbidities such as hypertension, diabetes and heart disease that may exert an impact on therapy efficacy (59, 60). In 2018, the costs of producing autologous cell therapies were estimated to be US$ 94 per million cells for a dose of 2 million cells per kg, which is calculated to be US$ 13,160 per dose for an average-weight adult, which raises the question of whether stem cell therapy would benefit only the better socio-economic group (61). Comprehensive clinical research is essential to establish a clear transplantation protocol, considering the timing, route, and dosage for optimal therapeutic outcomes. This includes addressing the technical challenges of cell tracking, survival, and integration and ensuring ethical practices and cost-effectiveness to make stem cell therapy a viable option for a broader patient population.

## Challenges in South Carolina

**Figure 3:**
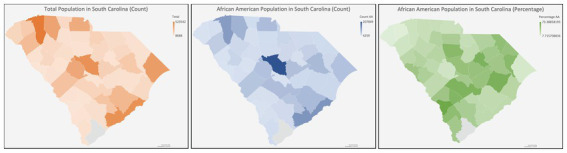
Diagram of South Carolina with the racial distribution.

South Carolina (SC) is characterized by a predominantly rural demographic, with approximately 35% of its inhabitants residing in rural locales, a figure substantially higher than the national average (62). Health disparities are more frequent in rural populations due to diminished prevalence of health insurance coverage, inferior socioeconomic and educational strata, and distinct cultural and societal influences (63) Because there are barriers to clinical trial enrollment in these areas (64), continued efforts to determine the obstacles to CT enrollment in SC regarding accessibility (e.g., lack of awareness, physicians not broaching CT options, unavailability of health insurance) and cognitive/psychological impediments (e.g., deficits in subjective and objective knowledge, prevalent misconceptions, ingrained distrust, apprehensions, and perceived risk) are needed (65) (Figure 3).

Several strategies can be applied to address enrollment barriers, including involving local physicians, community engagement/education, active recruitment, and financial incentives and support. A study monitoring community engagement with surgeons in the US Midwest found that most surgeons needed to be made aware of available trials and had no experience with the trial referral process (66). Furthermore, when later surveying patients following education, they described a more positive experience with their surgeons. This same study identified that facility communication and collaboration improved patient continuity of care. However, prior to seeing their referring physician, awareness of potential options extends to community and civic involvement (66). A study surveying 212 African Americans and Caucasians across rural and urban communities found that increased participation of churches/schools and family/friend referrals were more effective in rural communities versus in urban it was schools, media, and family/friends (67). This emphasizes the need to develop grassroots relationships in communities to foster a collaborative approach to medical access. This is further supported by another trial that identified low recruitment rates among rural and black individuals for palliative care clinical trials (68). In that study, recruitment strategies developed by community advisory groups aided in directing a more targeted approach to increase access and awareness of available trials. Of the 2,879 participants involved, 228 were eligible for potential trials. Of those who were enrolled in trials, only 12.7% consented when only a study coordinator was available, versus 58.8% when a community advisory group member was also present. This underlines the importance of embedded community allies in improving facility-community relationships. An analysis of recruitment strategies on pediatric RCTs in rural primary care clinics in 2022 found that utilizing traditional methods (i.e., posters, social media, press releases) was needed to complete enrollment for recruitment participation. In contrast, active enrollment (EMR-generated lists with staff follow-up) did (69). Furthermore, it reports that time to enrollment was quicker with active versus traditional methods. Lastly, financial barriers are among the most significant between rural and urban enrollment populations, as trials are ordinarily run in urban areas- thus requiring a considerable time commitment and financial commitment via travel, to participate in studies. Financial incentives alleviated these concerns and proved to be a significant motivator (67). Moreover, AAs exhibited a discernible gap in subjective and objective knowledge regarding CTs and an amplified perception of risk upon participation (65, 70). Gender-specific data pertinent to CT enrollment remains limited; however, one survey delineating gender-related discrepancies in CT willingness unveiled that females exhibited an increased perception of potential harm from trials ^despite displaying^ heightened susceptibility to financial inducements (71). Subsequent studies discerned that clinician trust and the perceived prospective benefits (either personal or altruistic) notably influenced females’ participation in CTs (72).

Addressing these cognitive and psychological barriers requires culturally sensitive education and awareness campaigns. These campaigns should focus on dispelling misconceptions, building trust, and providing clear information about the benefits and risks of clinical trials.

Enrollment volumes for stem cell therapy clinical trials vary between states in the United States, influenced by infrastructure, funding, and public awareness. States with established stem cell research centers and robust healthcare infrastructure tend to have higher enrollment volumes. For example, California, home to the California Institute for Regenerative Medicine (CIRM) and multiple Alpha Stem Cell Clinics, sees a high volume of patient enrollment. These centers provide a collaborative infrastructure that accelerates the development and validation of stem cell therapies, making California a leader in this field. Significant state-specific funding can impact enrollment. For instance, the UC San Diego Alpha Stem Cell Clinic received a grant from CIRM to expand clinical trials, highlighting the state’s commitment to advancing stem cell research and increasing patient enrollment. States with leading academic and research institutions, such as Massachusetts and Texas often have more robust public awareness and better enrollment rates in clinical trials. Rural states or those with less developed healthcare infrastructures need help enrolling patients due to logistical issues like transportation and limited access to specialized healthcare facilities. Addressing these barriers through local physician involvement and community engagement is essential for improving enrollment rates.

## Ethical considerations

In the United States, AAs have historically been subjected to experimental medical research while having limited access to quality healthcare (72). The unique socio-demographic landscape of SC, marked by its predominantly rural composition and higher-than-average AA population, necessitates a tailored approach to stem cell therapy research and application. It is not merely the impact of scientific innovation that must guide the research, but an acute understanding and acknowledgment of the existing health disparities that plague rural and AA communities in the state (73).The historical and systemic barriers these populations face, ranging from restricted access to healthcare, limited health insurance coverage, and socio-economic and educational challenges to deeply rooted cultural and societal norms, raise pivotal ethical questions (73).

Firstly, an ethical mandate is to ensure that the AA community is adequately represented in research trials, given its sizeable presence in SC. This is vital to ensure therapeutic efficacy and safety across the diverse genetic and socio-cultural landscapes. Beyond representation, the state also has higher rural demographics and inherent health disparities, emphasizing the need for equitable access. Given the intricate weave of socio-economic challenges, efforts must be undertaken to ensure that cost does not become a prohibitive barrier, particularly for the AA community and other marginalized groups in SC. Ethical considerations also extend to education and awareness campaigns. These campaigns must be culturally sensitive, addressing potential misconceptions and ensuring that the diverse populations of SC are informed and empowered to make decisions regarding stem cell therapies.

In conclusion, as SC navigates the promising terrain of stem cell research and application, it must do so with an ethical compass calibrated to its unique socio-demographic challenges. Only by doing so can the state ensure that the promise of stem cell therapies is a beacon of hope for all its residents, irrespective of race, socio-economic status, or geographical location.

## Author contributions

GM: Methodology, Resources, Writing – original draft, Writing – review & editing. DL: Software, Writing – original draft, Writing – review & editing. PG: Writing – original draft, Writing – review & editing. MR: Writing – original draft, Writing – review & editing. JV: Writing – review & editing.
