# Supplementary material for: Stem cell therapy: a new hope for stroke and traumatic brain injury recovery and the challenge for rural minorities in South Carolina
Source: Front Neurol. 2024 Aug 9;15:1419867. doi: 10.3389/fneur.2024.1419867 (PMC11342809; doi:10.3389/fneur.2024.1419867)
Supplement: Supplementary file 1 [file Table_1.DOCX]

Table (1): SCT Clinical trials in acute ischemic stroke and TBI

| Trial Reference | Country | Trial Phase | Administration route | Enrollment window | Number of participants | Outcome measures | Conclusion |
| --- | --- | --- | --- | --- | --- | --- | --- |
| Savitz, S.I. et al 2014 | USA | I | IV | 24-72 h | 8 | BI, mRS, NIHSS | Established safety, feasibility and good functional outcome |
| Hess, D.C et al 2017 | USA | II | IV | 24-48 h | 65 | mRS, NIHSS, BI | No difference for functional outcomes. |
| Laskowitz, D.T et al 2018 | USA | II | IV | 3–9 D | 10 | mRS, NIHSS | Established Safety |
| Friedrich, M.A. et al 2021 | Brazil | II | IA | 3–10 D | 20 | mRS, NIHSS | Established safety and potential role in better functional outcomes |
| Moniche, F. et al 2015 | Spain | II | IA | 5–9 D | 10 | mRS, BI, NIHSS | No difference in functional outcomes |
| Duma, C et al 2019 | UK | I | IA | 1 W | 5 | mRS, NIHSS | Established safety and potential role in better functional outcomes |
| Qiao, L.Y. et al 2016 | China | II | IV &IT | 1 W | 6 | NIHSS, BI, mRS | Showed some degree of neurological  recovery |
| Lu et al 2019 | USA | II | IV | < 12 m | 65 | 24 w FMMS | Improvement in motor function |
| Cox et al 2016 | USA | II | IV | <24 h | 25 | 21 d GCS, neurologic events | Radiologic improvement, modest clinical improvement, no adverse effects to infusion |

**Stem cells in stroke rehabilitation and recovery**

| **Trial Reference** | **Country** | **Trial Phase** | **Administration route** | **Enrollment window** | **Number of participants** | **Outcome measures** | **Conclusion** |
| --- | --- | --- | --- | --- | --- | --- | --- |
| Prasad et al 2014 | India | Phase II | IV | 7-30 days | 120 | Primary: BI and mRS  Secondary: NIHSS | No difference in outcome between groups  No difference in outcome between groups |
| Chen et al  2014 | China | Phase II | iv and stereotactic implantation | 6 months -5 years | 30 | Primary: NIHSS, ESS,EMS, mRS  Secondary: MRI-DTI, TMS-MEP | Improvement At 12 months,  positive MEP |
| Steinberg et al  2016 | USA | Phase 1/II | Stereotactic injection | 6 months - 60 months | 18 | Primary: safety  Secondary: ESS, NIHSS, FMS, mRS | Safe  Improvement in: ESS NIHSS, FMS no change in mRS |
| Kalladka et al  2016 | UK | Phase I | Stereotaxic ipsilateral putamen injection | 6-60 months | 11 | Primary: safety  Secondary: functional change (mRS, NIHSS, BI, MAS, EQ-5D) | Safe  Improvement in NIHSS |
| Levy et al  2019 | USA | Phase I/II | IV | >6 months | 15  21 | Primary: safety  Secondary: BI, BS, NIHSS, GDS, MMS | Safe  Improvement at 6 months |
| Muir et al  2020 | UK | Phase II | Stereotactic injection | 2-13 months | 23 | Primary: ARAT (≥2 points improvement)  Secondary: NIHSS, BI, FMS, MRS | Improvement in: 4% of patient at 3 months  Improvement at 12 months, ARAT (25%), mRS (35%), BI (40%) and FMS (30%) patients |
| Jaillard et al  2020 | France | Phase II | IV | <2 weeks | 31 | Primary: safety and feasibility  Secondary: NIHSS, BI, mRS, motor-NIHSS, motor-FMS | Safe, feasible  Improvement in:  Motor-NIHSS, motor-FMS |
| Chiu et al  2021 | China | Phase 1 | Stereotactic injection | 6 months -10 years | 6 | Primary: safety  Secondary: NIHSS, BI, BBS, FM-sensation | Safe  Improvement in NIHSS BI, BBS and FM-sensation |
